# Cellular and Molecular Mechanisms Underlying Liver Fibrosis Regression

**DOI:** 10.3390/cells10102759

**Published:** 2021-10-15

**Authors:** Alessandra Caligiuri, Alessandra Gentilini, Mirella Pastore, Stefano Gitto, Fabio Marra

**Affiliations:** Department of Experimental and Clinical Medicine, University of Florence, 50137 Florence, Italy; alessandra.caligiuri@unifi.it (A.C.); alessandra.gentilini@unifi.it (A.G.); mirella.pastore@unifi.it (M.P.); stefano.gitto@unifi.it (S.G.)

**Keywords:** liver fibrosis, fibrosis regression, myofibroblasts, HSCs, ECM degradation, therapies

## Abstract

Chronic liver injury of different etiologies may result in hepatic fibrosis, a scar formation process consisting in altered deposition of extracellular matrix. Progression of fibrosis can lead to impaired liver architecture and function, resulting in cirrhosis and organ failure. Although fibrosis was previous thought to be an irreversible process, recent evidence convincingly demonstrated resolution of fibrosis in different organs when the cause of injury is removed. In the liver, due to its high regenerative ability, the extent of fibrosis regression and reversion to normal architecture is higher than in other tissues, even in advanced disease. The mechanisms of liver fibrosis resolution can be recapitulated in the following main points: removal of injurious factors causing chronic hepatic damage, elimination, or inactivation of myofibroblasts (through various cell fates, including apoptosis, senescence, and reprogramming), inactivation of inflammatory response and induction of anti-inflammatory/restorative pathways, and degradation of extracellular matrix. In this review, we will discuss the major cellular and molecular mechanisms underlying the regression of fibrosis/cirrhosis and the potential therapeutic approaches aimed at reversing the fibrogenic process.

## 1. Introduction

**Figure 1:**
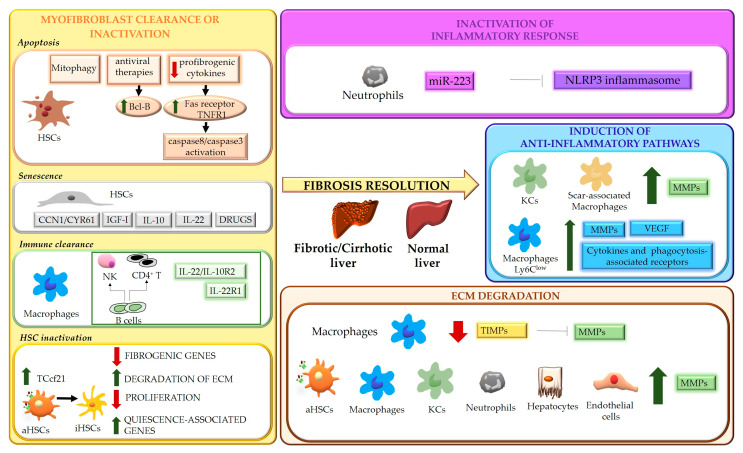
Schematic representation of the mechanisms underlying liver fibrosis regression. Four main mechanisms underlying the regression process of liver fibrosis are indicated. Hepatic stellate cells (HSCs); TNF receptor 1 (TNFR1); insulin-like growth factor I (IGF-I); transcription factor 21 (Tcf21); natural killer cells (NK); activated HSCs (aHSCs); inactivated HSCs (iHSCs); extracellular matrix (ECM); NOD-, LRR- and pyrin domain-containing protein 3 (NLRP3); matrix metalloproteases (MMPs); Kupffer cells (KCs); vascular endothelial growth factor (VEGF); tissue inhibitors of MMPs (TIMPs).

Chronic liver diseases caused by different agents may result in hepatic fibrosis, characterized by a sequence of events leading to excessive deposition of collagen and other extracellular matrix proteins, scar formation and altered liver structure and function, potentially conducting to organ failure in cirrhosis [1,2]. Although in the past years the fibrogenic process was considered a unidirectional and irreversible phenomenon, in the last decades reversal of fibrosis, upon removal of the damaging agent(s), has been described in several tissues. In the liver, due to its regenerative ability, the extent of fibrosis regression and restitution towards normal architecture is higher than in other tissues, even in advanced disease. In recent years, several clinical observations and experimental studies have improved the mechanistic understanding of the fibrogenic process, providing information on the molecular mechanisms underlying reversal of liver fibrosis. Currently, as reviewed in some articles [3,4,5] the basis of fibrosis resolution can be recapitulated in the following major points:

(1) Interruption or removal of detrimental agent(s) causing chronic hepatic injury [6];
(2) Elimination or inactivation of myofibroblasts [7];
(3) Inactivation of inflammatory response and induction of anti-inflammatory/“restorative” pathways [8,9];
(4) Degradation of extracellular matrix [10];

The mechanisms underlying the regression of fibrosis are summarized in Figure 1.

## 2. Materials and Methods

This is a non-systematic review article using the following electronic sources: PubMed, MEDLINE, Google Scholar, Ovid, Scopus, and Web of Science. We used the following single terms “regression of liver fibrosis”, “liver fibrotic process regression”, “reversibility of cirrhosis”, “cellular and molecular mechanisms of fibrosis reversion” or in combination search terms: “regression of fibrosis”, “liver”, “antifibrotic therapies”. We examined all the articles reporting in vitro research, animal models and human related data in English language (inclusion criteria) excluding papers with unavailable full text, abstracts, book chapters and articles published before 1990 (exclusion criteria). Finally, we evaluated supplementary references in papers examined in the first search round.

## 3. Removal of Causative Agent(s)

Clinical evidence has recently demonstrated that compensated cirrhosis caused by chronic HBV or HCV infection is reversible following viral suppression or eradication [11,12]. These findings indicate that removal of the causative agent not only leads to interruption of fibrogenic signals, but also induces fibrolytic/restorative pathways, resulting in regression of fibrosis. However, a certain fraction of patients does not regress, suggesting a potential involvement of genetic/epigenetic mechanisms [13].

In experimental studies performed in mice treated with CCl_4_ to develop a pre-cirrhotic stage of liver injury and then allowed to spontaneously recover upon toxin withdrawal, resumption of CCl_4_ exposure rapidly induced profibrogenic features in HSCs, indicating that an “epigenetic memory” can be induced in these and, possibly, other cells [14,15].

### Genetic/Epigenetic Signatures

Several genetic diseases predispose to liver fibrosis and in some cases to cirrhosis. These could potentially impair reversal of the fibrogenic process [16]. Many of the genes such as ABCB4, ASL, ALDOB, GBE1, SLC25A13, FAH, and SERPIN1 are highly expressed in the liver and therefore mutations of these genes mainly affect this organ [17]. Most genetic aberrations triggering cirrhosis appear in childhood and are the main cause of pediatric liver cirrhosis, apart from childhood obesity [18]. In addition to the genetic alterations leading to hepatic fibrosis in the childhood, mutations of the PNPLA3 gene represent a major predisposing factor in non-alcoholic fatty liver disease (NAFLD) patients [19]. PNPLA3, encoding for patatin-like phospholipase domain-containing protein 3, is mainly found in hepatocytes, adipocytes, and HSCs. PNPLA3 is endowed with triacylglycerol (TG) lipase and acylglycerol transacylase activities and the TG hydrolase function is reduced by about ∼80% in the presence of the I148M mutation/substitution [20,21]. The PNPLA3 I148M variant has been associated with steatosis, NAFLD, NASH, and hepatocellular carcinoma [20,22,23]. The loss of PNPLA3-mediated TG hydrolase activity in this variant is not sufficient to induce hepatic steatosis, since Pnpla3^−/−^ mice did not show fatty liver disease [24]. Therefore, other mechanisms may underlie the development of hepatic steatosis. In mammalian cells, PNPLA3 accumulation in lipid droplets [25,26,27,28] is regulated by fasting/feeding cycles, whereas the mutated PNPLA3 (148M) accumulates into lipid droplets evading ubiquitin and/or autophagy protein degradation [29,30,31]. BasuRay et al. showed that the excess of PNPLA3 into the lipid droplets induce per se fatty liver disease and depletion of the mutant form could resolve the excess of hepatic fat accumulation [30].

HSCs contain droplets of retinoic acid, which, by inducing retinoic acid receptor (RAR) expression, inhibits the fibrogenic process [32,33]. It has been observed that PNPLA3 mutations decrease the amount of retinoic acid in HSCs leading to the reduction of RAR-mediated control on fibrogenesis [34]. PNPLA3 might therefore represent a good therapeutic target to control NAFLD-associated fibrosis, steatosis, and disease progression [17].

Epigenetic modifications and changes in expression/activity of epigenetic enzymes regulate many processes involved in fibrosis development, including cell activation, response to injury, and immune reaction. Epigenetic changes, mainly represented by DNA methylation and histone post-translational modifications (mostly methylation and acetylation) act as dynamic modulators of HSCs, repressing or inducing genes and transcription factors implicated in the fibrogenic process, and influencing the response of HSCs to changes in the microenvironment. DNA methylation, as well as changes in methylation enzymes, has been associated with changes in transcription of genes involved in nucleotide metabolism, signaling pathways (e.g., Wnt [35,36]), cell proliferation and apoptosis (e.g., Pten [37]), extracellular matrix (ECM) synthesis/degradation (Actg2, Col4A1/2, Loxl1/2, Adamts9, matrix metalloprotease-MMP15 [38]). Of note, altered expression of enzymes that regulate DNA methylation DNA methyltransferases (DMNTs) and ten-eleven translocation methylcytosine dioxygenases (TET) has been observed in fibrotic livers from different animal models and in patients [35,39].

Epigenetic events may serve as adaptive mechanisms. In a pivotal study on multigenerational influences on hepatic fibrogenesis in rats, Zeybel et al. demonstrated that epigenetic adaptation to liver injury could be passed on to F1 and F2 progeny. This adaptation, which consists of a reduced number of myofibroblasts, decreased expression of TGF-β1 and increased expression of PPAR-γ, was mediated by changes in DNA methylation and histone acetylation. Moreover, DNA hypomethylation of the PPAR-γ promoter correlated with milder fibrosis in NAFLD patients [40].

A better understanding of the complex epigenetic mechanisms regulating the fibrogenic process could be helpful to identify epigenetic signatures as diagnostic/prognostic markers and to develop novel therapeutic strategies. Selective inhibitors of histone modifying enzymes (histone deacetylases (HDACs) and histone methyltransferases) have been shown to inhibit proliferation and to induce apoptosis in HSCs [41,42,43], to reduce fibrogenic factors, and to reverse myofibroblast differentiation in various organs [43,44,45,46,47,48].

## 4. Myofibroblast Clearance or Inactivation

The role of activated myofibroblasts in the development of liver fibrosis is well established. Different cell types can contribute to the myofibroblast population, including HSCs, portal fibroblasts, bone marrow-derived collagen producing cells (fibrocytes) and, possibly, parenchymal cells undergoing epithelial-mesenchymal transition (EMT) [49]. Although the origin of activated myofibroblasts may vary depending on the different etiologies of disease [50], HSCs can be considered their major source, as demonstrated by studies showing that HSCs depletion improves fibrosis in models based on both CCl_4_ intoxication and bile duct ligation [51]. Even in biliary fibrosis, where portal fibroblasts have been suggested to be the primary cell type initiating the fibrogenic response, giving rise to more than 70% of myofibroblasts, activation of HSCs becomes crucial after the initial phases [50].

During fibrosis regression, in response to a decrease of fibrogenic stimuli, the number of myofibroblasts drops, due to multiple mechanisms, that include restraint of activation, apoptosis, senescence, immune clearance, and reversal to a quiescent-like phenotype [14,15,52,53,54].

### 4.1. Limitation of HSC Activation

HSC activation is markedly influenced by changes in cellular microenvironment. Apart from soluble mediators, ECM components, matrix stiffness and interactions with neighboring cells, such as injured hepatocytes, immune cells (particularly macrophages), and activated sinusoidal endothelial cells, play an essential role in sustaining HSC/myofibroblast activation [55]. During recovery, changes in the microenvironment consequent to injury cessation contribute to create a milieu unfavorable to HSC activation. Moreover, high levels of intracellular energy are required by HSCs to retain their activated phenotype, and factors interfering with intracellular energy metabolism can hold HSCs in a less secretory and active state [56]. Along these lines, HSCs undergo a metabolic reprogramming during activation, consisting in induction of aerobic glycolysis and reduction of gluconeogenesis and lipogenesis. This metabolic rearrangement, mediated by Hedgehog (Hh) and hypoxia-inducible factor (HIF)1α, leads to an accumulation of lactate that further sustains the transactivation process, inducing the expression of proliferative and profibrogenic genes. Interestingly, the amount of stromal glycolytic cells was found to correlate with the severity of liver fibrosis in patients and experimental models. According to these findings, inhibitors of Hh signaling, HIF1α, glycolysis and lactate accumulation could be helpful to limit HSC activation and revert aHSCs to a quiescent state. Costunolide, a natural compound with anti-inflammatory, anti-oxidant and anti-tumor actions [57], has been recently shown to negatively modulate HSC activated phenotype, through inhibition of hexokinase 2, a rate-limiting glycolytic enzyme [58], which maintains glucose inside the cells as a source of energy metabolism [57]. Glutaminolysis is also essential to providing high levels of energy required by HSCs to maintain the activated phenotype [59]. Glutamine synthetase (GS), as well as other enzymes implicated in glutamine metabolism (glutaminase (GLS), aspartate transaminase (AST) and glutamine dehydrogenase (GDH)), was found to be upregulated during HSC activation both in vitro and in experimental fibrosis [59]. Accordingly, the expression of GLS, GDH1, AST1 and AST2 genes was significantly enhanced in the liver of fibrotic patients [60]. Hh and its downstream effectors, as Hippo, Yes-associated protein (YAP) and transcriptional co-activator with PDZ-binding motif (TAZ), are crucial for the increase in glutamine metabolism, as demonstrated by the fact that blocking Hh cascade with specific inhibitors (cyclopamine or verteporfin) reduced glutaminolysis, mitochondrial respiration, cell proliferation and collagen synthesis in HSCs [61].

### 4.2. Apoptosis

Apoptosis, a form of programmed cell death, regulates the balance of proliferating and dying HSCs during the fibrogenic process. This phenomenon contributes to reduce the amount of myofibroblasts but is not sufficient to restore the integrity of the liver tissue.

Clearance of HSCs is induced by the cytotoxic action of natural killer (NK) cells after the removal of the injurious agent [62], due to the increase of ligands of NK receptors such as MICA, NKG2D, and ULBP2 in senescent aHSCs. Moreover, during fibrosis resolution, increased collagen degradation by MMPs induces HSC apoptosis by activation of death receptor-mediated signaling, including Fas and TNFR-1 receptors, increase in pro-apoptotic proteins (e.g., p53, Bax, caspase 9), and decrease of anti-apoptotic proteins, including Bcl-2 [63,64].

In addition, in response to reduced levels of profibrogenic factors, the expression of Fas or TNFR1 and of the cognate ligands increases in HSCs, stimulating caspase 8/caspase 3 activation and apoptosis [62]. Tumor necrosis factor-related apoptosis inducing ligand (TRAIL) has been recently identified as an additional inducer of apoptosis in HSCs, both in experimental fibrosis [65] and in vitro [66], and its effect involves NF-κB and miR145 [67]. Additional mechanisms of programmed cell death in aHSCs are mediated by cyclooxygenase-2 (COX-2), which metabolizes the endogenous cannabinoid 2-arachidonoyl glycerol (2-AG) leading to the production of the pro-apoptotic prostaglandin D2-glycerol ester (PGD2-GE) [68]. On the other hand, caspase-9-dependent apoptotic pathways are elicited by increased expression of Bcl-2, Bax and p53 [62]. A recent report showed that mitophagy, a mechanism that eliminates damaged mitochondria to maintain mitochondrial homeostasis, is increased in HSCs during the regression of fibrosis, in parallel with enhanced apoptosis. Mitophagy contributes to apoptosis inducing an increase in Bcl-B, a member of the Bcl-2 family [69].

### 4.3. Senescence

Senescence is a passive and irreversible mechanism of cell death contributing to myofibroblast clearance during fibrosis regression [52]. Senescent cells stop proliferating due to cell-cycle arrest associated with telomere shortening [52] or other alterations, including chromatin modifications [70], DNA damage [70], oncogene activation, loss of tumor suppressors [70] and cellular stress, such as abnormal nutrient/O_2_ levels, altered ECM, oxidative stress [70,71,72]. Through a senescence-associated secretory phenotype (SASP), senescent myofibroblasts promote reversal of fibrosis preventing further proliferation of fibrogenic cells, upregulating ECM-degrading enzymes, and downregulating ECM proteins, including collagens [73]. Recruitment and function of immune cells involved in the clearance of activated HSCs, such as NK cells, is also involved in this process [52]. A variety of proteins, such as the matricellular protein CCN1/CYR61 [74], insulin-like growth factor I (IGF-I) and interleukin (IL)-10, IL-22 have been identified as inducers of senescence in aHSCs. This process is also stimulated by different drugs [75,76], such as the celecoxib derivative, OSU-03012 [77], nuclear receptor agonists (PPARγ, RAR and retinoic X receptor (RXR)) [70], or phytochemicals as curcumin, which promotes HSC senescence via PPARγ/p53 [78]. Soluble egg antigens (SEA) of schistosoma japonicum were also reported to induce senescence in activated HSCs via FoxO3a/SKP2/p27 [77].

### 4.4. Immune Clearance

Clearance of myofibroblasts is also triggered by the immune system. Besides macrophages, that can promote aHSC apoptosis [79], activated NK and liver-specific natural killer T cells induce rapid killing of HSCs, secreting a wide range of cytokines [80,81,82].

Both senescent and activated, but not quiescent, HSCs can be eliminated by NK cells through retinoic acid early inducible 1/natural killer group 2 member D (NKG2D)-dependent and TRAIL-dependent pathways [81], whereas NKT cells selectively target activated HSCs by release of IL-30 [83] and IFN-γ [84]. Li et al. recently showed that activated NK cells require the p38/PI3K/Akt pathways to promote TRAIL-induced cytolytic effects on aHSCs [85]. NK cells also induce HSC apoptosis through FasL [84], due to high Fas expression in aHSCs, and TRAIL [86], and restrain HSC activation via IFNγ release [87]. Of note, as the fibrogenic process progress, NK cell activation tends to diminish, impairing the protective function of this system [88].

CD4^+^ T (Th1, Th2, Th17) and regulatory T cells (Treg) are major modulators of immune response, with direct or indirect effects on fibrosis regression [89]. Activated T lymphocytes were reported to induce aHSC senescence through IL-22/IL-10R2 and IL-22R1 [90]. γδT cells, that represent 3–5% of liver lymphocytes, were recently shown to promote fibrosis regression. Using a CCl_4_-induced model of fibrosis in γδT cell-deficient mice, Liu et al. demonstrated that these cells suppress liver fibrosis by at least two mechanisms, a direct cytotoxic effect on aHSCs, triggered by NKp46, and an indirect action, involving the crosstalk with NK cells. The IFN-γ releasing subset (γδT1) was found to be more active against aHSCs than the IL-17 secreting subtype (γδT17) [91].

The role of B lymphocytes in hepatic fibrogenesis is mediated by direct or indirect actions on different cell types, including HSCs, NK cells or CD4^+^ T lymphocytes [83]. Faggioli et al. showed that in a mouse model of chronic fibrosing cholangitis, ablation of B lymphocytes and consequent downregulation of the TNF-α/NF-κB pathway suppresses HSC activation and induces HSC senescence [92].

### 4.5. HSC Inactivation

Development of promoters selectively driving transgenes in HSCs, to achieve cell-specific gene expression, revealed that aHSCs can revert to an inactive/quiescent-like phenotype during regression of liver fibrosis [14,15]. In an elegant study performed using the Cre-LoxP-based genetic labeling technique, Kisseleva et al. investigated the fate of HSCs in alcohol- or CCl_4_-induced experimental fibrosis. During recovery from fibrosis, about 50% of hepatic myofibroblasts escape apoptosis and revert to a quiescent-like phenotype, downregulating fibrogenic genes and upregulating the survival factors Hspa1a/b [14]. An interesting study by Song et al. showed that upon ectopic expression of the transcription factors FOXA3, GATA4, HNF1A, and HNF4A, mouse myofibroblasts transdifferentiate into hepatocyte-like cells. The transcriptional reprogramming was achieved both in vitro and in vivo, in fibrotic mice, resulting in amelioration of liver fibrosis [93]. Recently, transcription factor 21 (Tcf21) has also been identified as a deactivation factor for myofibroblastic HSCs. TCcf21 levels decrease during the fibrogenic process both in humans and mice and return to normal levels upon regression of murine fibrosis. TCf21- overexpressing aHSCs reverted to a quiescent phenotype with consequent regression of fibrosis and amelioration of hepatic structure and function. Of note, HSCs overexpressing TCf21 failed to store vitamin A, indicating that this transcription factor is unable to modulate the whole program of HSC deactivation [94].

The above findings are in agreement with in vitro studies showing that aHSCs can revert to a quiescent-like status, acquiring a novel phenotype, similar but distinct from the original quiescent cells, and characterized by low proliferation rate and elevated metalloproteinase activity [95,96]. Gene expression analysis revealed that inactivated (i) HSCs display reduced levels of fibrogenic genes such as collagens, LOX and α-smooth muscle actin (α-SMA), and increased expression of adipogenic, quiescence-associated genes, such as PPARγ. Of note, the expression of glial fibrillary acidic protein, adiponectin receptor1, Adpf, and D site of albumin promoter binding protein, typical of the quiescent status of HSCs, remain absent [96]. Functionally, iHSCs are more sensitive to fibrogenic stimuli and rapidly reacquire profibrogenic features [14]. Interestingly, in vitro studies performed on a gradually softening hydrogel mimicking microenvironmental changes occurring during fibrosis progression and regression, proved that mechanical stimuli are crucial for activation and reversion of HSCs [97]. Accordingly, a recent study by Dou et al. showed that substrate stiffness in vitro or liver stiffness in vivo induced post-translational changes in histones, transcription factors and coactivators in HSCs, leading to their activation. These events were mediated by the histone acetyltranferase p300. Indeed, stiffness induced, via RhoA/Akt, the phosphorylation and nuclear translocation of p300, resulting in transcription of several genes associated with the HSC profibrogenic phenotype, as α-SMA, CTGF, PDGFA and B, VEGFA, IL-11, IL-6, CXCL12 [98]. From a translational point of view, these findings suggest the possibility to induce fibrosis regression by affecting specific signals that trigger this response [99].

## 5. Modulation of Inflammatory Processes

Inflammation represents a main feature of chronic liver diseases and plays a key role in any stages of the fibrogenic process, even during fibrosis regression. Inflammatory response involves multicellular interactions, dynamically regulated by a plethora of factors (e.g., soluble mediators, ECM components, pathogen-associated molecular patterns-PAMPs, damage-associated molecular patterns-DAMPs), acting in cell-specific fashion and aimed to restore liver architecture and function, but also leading to liver fibrosis when the noxious agent persists.

Cell death is an early and primary inducer of chronic inflammation and fibrosis. Hepatocyte-derived apoptotic bodies stimulate the secretion of pro-inflammatory and profibrogenic cytokines from macrophages and promote activation of HSCs through induction of autophagy [100,101,102]. In addition, injured hepatocytes release DAMPs, such as ATP, phormyl peptides, High Mobility Group Box 1(HMGB1) [103] and cytokines such as IL-33 [104], which triggers HSC activation directly or indirectly, by promoting IL-13 release by innate lymphoid cells (ILC2). At the same time, inflammatory mediators secreted by infiltrating immune cells contribute to cell death, amplifying hepatic injury [104].

As major effectors of fibrosis, activated HSCs play a central role in inflammation, receiving a wide variety of stimuli from inflammatory cells and from hepatocytes, cholangiocytes and activated sinusoidal endothelial cells (SECs). Activated HSCs are highly responsive to inflammatory mediators which induce inflammatory pathways (such as NF-κB and AP-1) [105,106] and consequent secretion of cytokine/chemokines that act in autocrine and paracrine fashion. Inflammatory signals exert specific roles on HSCs, maintaining survival (IL-1β, TNFα, CXCL12) and the activated state (ILs and chemokines) [107], providing chemotactic stimuli for HSCs themselves or inflammatory cells (CCL2, CCL5, CXCL9, CXCL10, CX3CL1) and mediating the gut-liver axis crosstalk (toll like receptors (TLRs)) [105]. All these processes can contribute to positively or negatively modulate inflammatory responses and fibrogenesis, promoting fibrosis progression or regression.

As modulators of liver fibrosis, immune cells exhibit a dual role, being able to contribute to both fibrosis progression and regression [108,109]. Danger signals generated in the site of injury lead to infiltration of circulating inflammatory cells (T lymphocytes, neutrophils, dendritic cells and monocytes) and activation of Kupffer cells (KCs) [108,109]. The release of a wide range of soluble mediators amplifies inflammation and stimulates the fibrogenic process. Upon removal of the cause of injury, the balance switches from pro- to anti-inflammatory/restorative pathways, promoting fibrosis resolution. This shift is achieved by rearrangements in the type of immune cell populations recruited, with a marked drop in intrahepatic T cells and blood-derived cells (NKT cells, monocytes) [110], and phenotypic modifications of certain cell types, mainly macrophages.

### 5.1. Neutrophils

Neutrophils have been recently shown to play an important role in the resolution of the inflammatory response in various tissues [111,112,113]. Mice with neutrophil depletion during the recovery phase of liver inflammation showed impaired hepatic fibrosis and altered liver architecture. A similar outcome was observed in mice with deletion of the granulocyte-specific miR-223 gene, a negative post-transcriptional regulator of NLRP3 inflammasome. A complete recovery of liver function could be achieved restoring miR-223 levels or with adoptive transfer of wild-type neutrophils. These findings indicate a potential restorative phenotype of neutrophils expressing miR-223, able to promote the resolution of the inflammatory process [114]. High polymorphonuclear (PMN) cell infiltration in liver biopsies of patients with alcoholic hepatitis has been associated with better prognosis, further supporting a regenerative function of neutrophils, and suggesting that sustaining liver regeneration could be more appropriate than inhibiting the inflammatory process [115].

### 5.2. Macrophages

Hepatic macrophages derive from both circulating monocytes, recruited to the injured liver via growth factors and chemokine signals, or from self-renewing embryo-derived resident macrophages, called KCs [116]. Macrophages are a highly plastic and heterogeneous population with multiple functions, according to injury kinetics and environmental settings. Recent reports on single-cell mRNA sequencing of liver cell populations highlighted the heterogeneity and plasticity of the macrophage compartment in both rodent models and human disease [116,117,118]. Macrophage heterogeneity and plasticity are characterized by different cell surface markers and transcriptional profiles, and different stimuli can induce the polarization of macrophages [119]. In most cases, a classification in two main subsets: M1 (classically activated) and M2 (alternatively activated) is widely used [120].

Classically activated macrophages differentiate into M1 macrophages producing pro-inflammatory cytokines such as IL-6, TNF-a, IL-1, IL-12, IL-15, and IL-18 [121], whereas alternatively activated macrophages modulate inflammatory reactions and mediate tissue repair. Alternatively activated macrophages can be further distinguished in diverse subtypes, each induced by different molecules and eliciting different signals. In particular, M2a macrophages are stimulated by IL-4 and IL-13, and mainly induce a Th2 response. M2b macrophages are stimulated by immune complexes and are involved in Th2 activation and immune regulation, and M2c macrophages are stimulated by IL-10 or TGF-β and are involved in immune suppression, tissue repair and matrix remodeling [119]. However, this traditionally classification based on induction of in vitro polarization does not well describe the phenotypic heterogeneity of hepatic macrophage in vivo [109]. In murine models, Ly6c expression is used to characterize populations of circulating monocytes and macrophages in pathology [119,122]. Circulating Ly6c^+^/^high^ and Ly6c^−^/^low^ monocytes have been well characterized. Their counterparts in humans are classically activated (CD14^+^ CD16^+^) monocytes expressing CCR2, CD64, and selectin L and non-classical (CD14^+^ CD16^−^) monocytes which do not express CCR2, respectively [123]. Ly6c ^high^ monocytes are considered precursors to Ly6c^high^ and Ly6c^low^ macrophages. Ly6c^high^ mirror M1 macrophages exhibiting pro-inflammatory phenotype, while Ly6c^low^ macrophages exhibit an M2-like phenotype that play an anti-inflammatory role during liver damage [124].

In the healthy liver the number of KCs remains constant, and they are the predominant macrophage population in the liver; following liver damage the intrahepatic macrophages are massively expanded, due to the influx of peripheral monocytes [125].

#### 5.2.1. Embryologically-Derived/Resident Macrophages

KCs express specific markers useful for their characterization, such as F4/80, CD11b^+/low^, CD68 and, C-type lectin domain family 4 member F (CLEC4F) in mice [122]. In the early stages of liver damage, KCs exert proinflammatory and protective actions, through the release of cytokines and chemokines, which further recruit other immune cells. Simultaneously, KCs play a relevant role in the fibrogenic process, via TGF-β and PDGF-mediated activation of HSCs [81]. After removal of injury, hepatic macrophages contribute to fibrosis resolution by secreting MMPs [15,95]. In addition, they also interact with NKT cells that, as reported above, contribute to aHSC elimination [81].

#### 5.2.2. Bone-Marrow/Monocyte-Derived Macrophages

During injury, activated KCs and HSCs induce the recruitment of Ly6C^hi^ expressing monocytes, which rapidly convert to Ly6C^hi^ macrophages characterized by high phagocytic activity [126], through CCL2/CCR2 and other chemokine systems. These cells can secrete a variety of mediators (e.g., TNF, IL-6, IL-1β or TGF-β) that can act in a proinflammatory or anti-inflammatory/profibrogenic fashion, depending on the timing of release and the immune/ECM microenvironment [79,124].

During fibrosis regression, macrophages undergo phenotypic conversion to a restorative Ly6C^low^ subset, able to secrete MMPs like MMP9 and MMP12, growth factors such as VEGF and cytokines, and express phagocytosis-associated receptors [124]. This shift is induced by phagocytosis of apoptotic myofibroblasts and/or injured hepatocytes and is mediated by the fractalkine receptor CX3CR1 [127]. As mentioned above, MMPs secreted by restorative macrophages may vary during the regression process, being influenced by soluble mediators released in the milieu. Thus, VEGF-induced CXCL9 release in macrophages results in MMP13 secretion [128], whereas the IL-4/IL-13/IL-4Rα axis mainly stimulates MMP2, through STAT6 [129]. Interestingly, IL-4Rα is a key player in macrophage polarization toward the anti-inflammatory/restorative M2 phenotype but is also involved in hepatic inflammation and fibrosis during the fibrogenic process [129].

In cirrhotic patients, [130], Cardoso et al. observed an increase in circulating intermediate monocytes (CD14^+^ CD16^+^), distinguished from the classical monocytes (CD14^++^ CD16^−^) and from nonclassical monocytes (CD14^−/low^ CD16^+^) [131]. They detected alterations in the proportions of circulating monocytes, particularly in patients with more advanced liver disease. Moreover, the cytokine profile analyzed in this study showed elevated plasma levels of IL-6 and IL-10, particularly in patients with acute decompensation of cirrhosis. Taken together, these findings indicate in cirrhosis the presence of systemic effects that influence the immune–hematopoietic system. Interestingly, a distinct population of scar-associated macrophages deriving from recruitment and differentiation of circulating monocytes has been identified, following liver damage. This macrophage subtype has an important role in resolution of liver fibrosis, representing one of the sources of MMP13 in fibrotic niches in livers of cirrhotic patients [132]. During the early stages of liver injury, scar-associated macrophages differentiate into inflammatory macrophages, and subsequently switch to an anti-inflammatory phenotype, which secretes a wide variety of MMPs to facilitate fibrosis resolution [133,134].

## 6. ECM Degradation

Liver fibrosis is a dynamic process characterized by an unfavorable balance between ECM deposition and degradation. Degradation of ECM represents one of the most relevant aspects of fibrosis regression and requires activation of MMPs, macrophage phagocytic activity and downregulation of MMP-inhibitory molecules, such as tissue inhibitors of MMPs, TIMPs [7,10]. MMPs are the main matrix-degrading enzymes [62] and, according to substrate specificity, can be grouped in collagenases (MMP8, MMP1 and MMP13) which cleave native fibrillar collagens to gelatin, gelatinases (MMP2, MMP9), degrading a wide range of substrates including gelatin, collagens and, in some extent, elastin, metalloelastases (MMP12) and others (Table 1).

They are secreted by various cell types, including aHSCs, hepatocytes, endothelial cells, and inflammatory cells, such as neutrophils and macrophages. MMP release and activity are finely regulated during the different phases of fibrogenic process, as well as during fibrosis regression. In this context, a relevant role in matrix degradation is played by “restorative” macrophages that, besides a role in phagocytic digestion of matrix fragments, represent a major source of MMP12, MMP13, and MMP9 [124]. In a recent study, Feng et al. showed that in thioacetamide (TAA)-induced fibrosis, depletion of KCs delayed resolution following toxin withdrawal and this was mainly ascribed to a marked decrease in MMP9 [135]. Because activated HSCs display high TIMP levels, ECM degradation strictly correlates with HSC clearance and the subsequent shift in MMPs/TIMPs balance, creating the conditions for a milieu favoring parenchymal regeneration.

## 7. Reversibility of Cirrhosis

ECM remodeling is crucial in determining reversibility of fibrosis. In recent years, clinical and experimental studies have provided evidence that matrix remodeling and at least partial restitution towards a normal architecture may be observed even in advanced liver fibrosis or cirrhosis [136,137,138,139,140]. The amount of elastin and cross-linked proteins in fibrotic scars is critical in this process. Protein cross-linking, which is mediated by cellular transglutaminases and lysyl oxidases, stabilizes ECM, enhancing its resistance to enzyme degradation and, together with elastin, increases matrix stiffness, that further sustains HSC activation via integrin-mediated mechanisms [62]. In this setting, MMP12 released by macrophages can still promote matrix turnover acting not only on elastin but also on collagens [141]. However, ECM remodeling in cirrhotic scars is also influenced by vascular remodeling that can hamper matrix degradation [142]. Thus, even when restitution to normal liver architecture is achieved, cirrhosis-associated derangements in the vascular system and in other organs persist. By using two different models of cirrhosis induction and reversal (TAA and BDL), Hsu et al. demonstrated that, despite a complete regression of fibrotic scars, portal hypertension was only partially reduced, due to persisting alterations in splanchnic and collateral circulation [143].

These biologic considerations have clear clinical implications. Regression of fibrosis represents a major clinical goal, since it can lead to a recovery of liver function and reduction in portal pressure, which decrease the incidence of portal-hypertensive complications and of hepatocellular carcinoma (HCC) [144,145,146,147]. It is well known that mild and moderate fibrosis can be reversible, but the same concept is not always true for cirrhosis. In this respect, the identification of a “point of no return” in the natural history of liver disease can be very difficult, despite its utmost relevance in clinical practice. This may be viewed as a condition beyond which even causal therapy (e.g., viral eradication) does not determine a significant regression of fibrosis and/or has limited impact on the appearance of complications and prognosis of the patient. As indicated above, the degree and amount of structural damage, in particular the development of extensive matrix crosslinking [148] and accumulation of elastin fibers in long-standing cirrhosis, have been indicated as a major element to identify the “point of no return” [141].

From a clinical standpoint, the ‘model’ of HCV eradication has provided relevant data in this context. Patients with compensated cirrhosis (Child-Turcotte-Pugh class A) achieving viral eradication with direct-acting antivirals (DAAs), show regression of fibrosis in a relevant percentage of cases (88%) [149] and a consequent decrease of portal hypertension [150]. When patients with cirrhosis Child-Pugh class B and C are considered, long-term data about the effects of sustained virologic response (SVR) after DAA treatment on fibrosis and liver-related complications and survival are less abundant. However, data from other contexts (e.g., HBV or alcohol-related decompensated cirrhosis) indicated that Child C class could represent a “point of no return” in terms of fibrosis decrease even after removal of the etiologic factor [151,152]. Other clinical predictors of the lack of fibrosis regression include age (>65 years), albumin (<3.5 g/dL), high MELD score (>20), alcohol habit and presence of metabolic disorders. However, none of them are satisfactory consistent to be used in clinical practice [153].

Advanced liver fibrosis and cirrhosis are major risk factors for HCC [154,155]. In particular, fibrosis and cancer-associated fibroblasts (CAF,) can influence the onset of HCC modulating the cancer microenvironment [156,157]. Considering these assumptions, HCV eradication should determine a decrease of both HCC occurrence and recurrence. In recent years, this has been a very debated issue since some studies suggested that SVR due to DAA, differently from interferon-based therapies, could increase the risk of both occurrence and recurrence of liver cancer [158]. It is now accepted that there is no such risk on a population basis and, as recently demonstrated [159], SVR due to DAAs leads to a drop in all-cause mortality, hepatic decompensation, and HCC. Nevertheless, on an individual basis, DAAs might favor the HCC development in subjects who already have a predisposing hepatic condition such as activated neo angiogenesis [160]. Moreover, subjects with severe metabolic impairment may have a risk of HCC despite viral eradication [161]. DAA-induced modifications in VEGF, epidermal growth factor, and inflammatory factors have been proposed for the detection of subgroups at risk of HCC, and some authors have proposed these as possible determinants of the susceptibility to cancer development [162,163].

## 8. Vascular Remodeling

As anticipated above, angiogenesis and vascular remodeling represent additional mechanisms involved in both fibrosis development and regression. Although the role of angiogenesis in promoting liver fibrosis is fully accepted, new lines of evidence indicate that angiogenic factors may also induce scar degradation and tissue repair during fibrosis resolution. Using murine models of fibrosis reversal, Yang et al. showed that the VEGF/VEGFR2 pathway is essential to maintaining sinusoidal permeability and the subsequent monocyte infiltration and macrophage fibrinolytic activity [128]. Moreover, VEGF release by macrophages was shown to be critical for fibrosis resolution. In fact, VEGR2-mediated activation by VEGF induced ECM degradation through upregulation of MMPs and downregulation of TIMPs in sinusoidal endothelial cells [164].

Capillarization of the sinusoids and changes in liver sinusoidal endothelial cells (LSECs) represent key events in liver fibrogenesis, triggering HSC activation and impairing hepatocyte polarization. These consist in LSEC dedifferentiation with loss of fenestrae and deposition of a continuous basement membrane that hampers normal exchanges between blood circulation and hepatocytes. Restoration of differentiated LSEC is crucial for recovery from hepatic fibrosis, as proved by the fact that depleting factors implicated in sinusoidal permeability, such as VEGF or CXCL9, results in delayed recovery [128]. In a thioacetamide-induced rat model of cirrhosis, administration of BAY 60-2770, an activator of soluble guanylate cyclase (sGC), promoted a complete reversal of sinusoid capillarization, by restoring normal levels of cGMP, fenestrae, and porosity in LSECs. Restitution to differentiated LSECs resulted in reversal of HSC activation and regression of fibrosis. Moreover, maintenance of physiological levels of cGMP in LSECs was essential to prevent fibrosis progression [165]. Liver X Receptor (LXR) α, which mediates multiple antifibrogenic actions interfering with the activation of HSCs, the release of inflammatory mediators and the synthesis of profibrogenic factors [56,166,167,168], was hypothesized to play a role in reverting capillarization of the sinusoids, through inhibition of Hedgehog-dependent signaling in LSECs [169]. In a mouse model of biliary fibrosis induction and reversal, Lee et al. identified AKAP12, a scaffold protein expressed in various cell types regulating cyclic adenosine monophosphate (cAMP) compartmentalization, as a novel mediator of fibrosis resolution, through mechanisms affecting LSEC dedifferentiation/activation and angiogenesis [170]. In an elegant study, Xu et al. identified leukocyte cell-derived chemotaxin 2 (LECT2) as a ligand of Tie1 (an orphan receptor expressed by endothelial cells) and LECT2-Tie1 as a novel profibrogenic pathway involved in vascular remodeling, that enhances sinusoid capillarization and reduces portal angiogenesis. They showed that knockdown of LECT2 (in both LECT2 KO mice and AAV9-LECT2 shRNA- treated mice) attenuates fibrosis development and ameliorates established fibrosis in different experimental models, reducing sinusoid capillarization and increasing portal angiogenesis. Notably, serum levels of LECT2 were significantly increased in patients with advanced fibrosis, indicating LECT2-Tie1 signaling as a promising therapeutic target [171].

## 9. Potential Strategies to Accelerate Fibrosis Reversal in Preclinical and Clinical Studies

It is well known that liver fibrosis and even cirrhosis may reverse after removing the underlying chronic disorder. This concept is consolidated for subjects with controlled hepatitis B virus replication and for patients with chronic hepatitis C infection achieving sustained virological response, while solid evidence for patients with alcoholic and non-alcoholic steatohepatitis is still lacking [148,172,173,174]. Consequently, a deeper knowledge of the mechanisms underlying fibrosis regression is needed to develop potential therapeutic approaches.

### 9.1. Targeting ECM Remodeling and Sinusoidal Permeability

Targeting ECM remodeling represents an effective strategy. Induction of macrophage-mediated ECM degradation via MMPs may be helpful. Feng et al. demonstrated that in a mouse model of liver fibrosis, resolution was delayed by KC depletion and accelerated by adoptive transfer of KCs from WT animals, compared to KCs from MMP9^−/−^ mice, suggesting that KC-derived MMP9 is essential in fibrosis reversal [135]. Selective lysyl oxidase-like 2 (LOXL2) inhibitors reduce ECM stabilization and resistance to MMP degradation by interfering with collagen and elastin cross-linking [175]. However, targeting LOXL2 in clinical trials with humanized anti-LOXL2 has shown no clinical benefit so far [176,177]. In order to reduce collagen 1 (Col1), Hsp47, a Col1 chaperone, was blocked in models of liver fibrosis by Hsp47 siRNA contained in vitamin A-coupled liposomes, which are predominantly taken up by HSCs, reporting anti-fibrotic actions [178]. A trial conducted with an HSP47 siRNA delivering lipid nanoparticle did not show any toxicity in healthy subjects (Soule B. et al. safety, tolerability, and pharmacokinetics of BMS-986263/ND-L02-s0201, a novel targeted lipid nanoparticle delivering HSP47 siRNA, in healthy participants: a randomized, placebo-controlled, double-blind, phase 1 study-unpublished raw data). Negative modulators of LSEC dedifferentiation and activation, such as LXRα agonists, that also display other protective actions could be effective to revert LSEC capillarization, a prerequisite for fibrosis resolution [169,170].

### 9.2. Agents That Reduce the Activation of HSCs

cAMP, a second messenger involved in several cellular responses, has been shown to promote fibrosis regression and could be a potential target to slow down fibrosis [179]. High levels of cAMP inhibit the activation of HSCs and fibroblasts, reduce their proliferation and survival, and decrease ECM synthesis [180,181]. Cilostazol is a semi-selective inhibitor of phosphodiesterase III, which increases intracellular cAMP leading to increased concentrations of the active form of protein kinase A (PKA) [182]. The use of this drug was first approved by the FDA as a treatment for intermittent claudication in 1999 [183], and it has been studied in other clinical settings displaying pleiotropic biomolecular mechanisms, including platelet inhibition, vasodilation, anti-proliferation, neuroprotection and reduction of ischemic-reperfusion injury [184,185,186,187,188]. This agent has also displayed antifibrotic actions in experimental nonalcoholic fatty liver disease [189] and was shown to suppress HSC activation, reducing CCl_4_-induced liver fibrosis [190]. Recently, cilostazol was reported to promote fibrosis regression in a TAA-induced model, through the up-regulation of hepatic cAMP and modulation of inflammation, oxidative stress, and apoptosis [191]. Amelioration of fibrosis was also observed in an alcohol-induced rat model, in which cilostazol decreased α-SMA, collagen I and III, TGF-β1 and connective tissue growth factor (CTGF) expression [192]. These results suggest that cilostazol could be a potential anti-fibrotic agent, although further studies are necessary to better understand its mechanisms of action. Moreover, despite its many beneficial effects, the treatment of patients with this drug must be cautious. Due to their vasodilator properties, in patients with class III to IV heart failure phosphodiesterase inhibitors have been associated with reduced survival compared with placebo. In addition, patients with history of ischemic heart disease could have a higher risk for worsening of angina pectoris or myocardial infarction [193]. Cilostazol contraindications may include tachycardia, tachyarrythmia, and/or hypotension [194]. Therefore, attention is necessary in prescribing cilostazol to patients affected by atrial or ventricular ectopy and/or by atrial fibrillation or flutter [195].

Activated HSCs increase their contractile properties in response to endothelin-1 (ET-1) via autocrine mechanisms and paracrine crosstalk with LSECs and damaged hepatocytes [196]. A current clinical trial is examining the potential effect of an ET-1 receptor inhibitor in patients with cirrhosis (NCT03827200). It is well known that the renin-angiotensin system is up-regulated in cirrhotic livers [197,198]. Agents targeting this system are effective to reduce TGF-β1 levels and the extent of fibrosis, with good safety outcomes, in fibrotic patients [199]. An undergoing clinical trial is testing the angiotensin receptor blocker, candesartan, in cirrhotic patients (NCT03770936).

Other targets include the Wnt/-catenin signaling, which plays an important role in HSC activation driving to liver fibrosis [200,201,202]. PRI-724, a cyclic AMP-response element binding protein (CBP)/beta-catenin inhibitor, has been observed to inhibit HSC activation and collagen production in HCV transgenic mice [203]. In addition, in a CCl_4_ murine model of liver fibrosis, PRI-724 ameliorated fibrosis owing to an increase in of F4/80^+^ CD11b^+^, and Ly6C^low^ CD11b^+^ macrophages [124]. The CBP/beta-catenin-dependent mechanism of action of PRI-724 was well outlined in CBP KO mice [204]. A phase I clinical trial with PRI-724 conducted in HCV-associated liver cirrhosis patients showed a dose dependent histological improvement only in a few patients. NCT03620474, a phase I/II clinical trial with PRI-724 in HCV or HBV- associated liver cirrhosis patients, will be completed in 2022 and will further clarify the safety and effectiveness of PRI-724 as anti-fibrotic agent.

Farnesoid X receptor (FXR), a bile acid activated nuclear receptor mainly expressed in liver and intestine, is a key regulator of hepatic bile acid homeostasis, lipoprotein, and glucose metabolism, inflammatory responses, and liver regeneration [205,206,207]. FXR has been shown to exert inihibitory effects on HSCs activation [208,209,210]. Obeticholic acid (OCA), the first small molecule to target FXR to be approved by FDA in 2016 as a second-line treatment for primary biliary cholangitis [205], has also been used in clinical trials of patients with fibrosis. In a 2015 phase II clinical trial study (FLINT), a short-term treatment with OCA (72 weeks) improved fibrosis in NASH patients [211]. In a recent phase III clinical trial (REGENERATE) with long-term OCA treatment in NASH patients (NCT02548351) obeticholic acid 25 mg significantly improved fibrosis and key components of NASH disease activity among patients with NASH [212].

Hepatocyte cell death triggers HSC activation [213]. The pan-caspase apoptosis inhibitor emricasan has been used in preclinical [214] and clinical studies [215,216]. While in a fibrotic rat model this agent ameliorated portal hypertension and liver fibrosis [214], the clinical trials conducted so far have not been successful [215,216]. Another apoptosis inhibitor tested as anti-fibrotic agent is selonsertib. This agent inhibits the activation of signal–regulating kinase 1 (ASK1), a serine/threonine signaling kinase, implicated in the activation of stress response pathways that exacerbate hepatic inflammation, apoptosis, and fibrosis (Budas G et al. reduction of liver steatosis and fibrosis with an ASK1 inhibitor in a murine model of NASH is accomplished by improvements in cholesterol, bile acid, and lipid metabolism -unpublished raw data). In a murine model of NASH, selonsertib significantly ameliorated not only metabolic parameters associated with NASH but also decreased hepatic steatosis, inflammation and fibrosis and in a DMN-induced fibrosis rat model it could reduce collagen deposition and the expression of α-SMA, fibronectin, and collagen type I (Budas G et al. reduction of liver steatosis and fibrosis with an ASK1 inhibitor in a murine model of NASH is accomplished by improvements in cholesterol, bile acid and lipid metabolism -unpublished raw data), [217]. Based on a successful phase II clinical study enrolling NASH patients [218], two phase III clinical trials have examined the safety, and anti-fibrotic efficacy of selonsertib in NASH patients with bridging fibrosis (STELLAR-3 trial) or compensated cirrhosis (STELLAR-4 trial). While selonsertib did not show any adverse effects both trials failed to reach the primary endpoint of fibrosis improvement at week 48 [219].

### 9.3. Therapeutic Targeting of Hepatic Macrophages

There are different possible approaches for targeting hepatic macrophages for the treatment of liver diseases, including the reduction of circulating monocyte recruitment, the inhibition of KC activation and the modulation of macrophage polarization [220]. As previously mentioned, recruitment of proinflammatory monocytes in the injured liver is mediated by chemokines secreted by various activated liver cells that mediate a chemotactic action. Among therapeutic approaches, the modulation of chemokine signaling models using monoclonal antibodies, receptor antagonists, aptamer molecules and small molecule inhibitors has proven efficacy in various experimental models, as reported in the session of this review dedicated to CC chemokine receptor antagonists.

Another possible targeting strategy to treat liver diseases is to modulate KC activation, e.g., acting on surface molecules on KCs that have an important role in the fibrogenic process [221]. In rodent models it has been observed that galectin-3 inhibitors (GR-MD-02 and GM-CT-01) significantly decreased septal galectin-3 positive macrophages with reduction of fibrosis levels and portal pressure [222]. Galectins are carbohydrate-binding proteins in glycoproteins components of the ECM. Galectin-3 is highly expressed on KCs and plays a vital role in cell adhesion, inflammation, and fibrogenesis. GR-MD-02, a galectin-3 inhibitor, is safe and well-tolerated in subjects who had a definite histological diagnosis of NASH with advanced fibrosis, and these data provided support for a development program in advanced NASH fibrosis [223]. Recently, a phase II clinical trial of GR-MD-02 was conducted in 162 patients with NASH, cirrhosis, and portal hypertension. Although levels of fibrosis, NAFLD activity scores and liver-related outcomes did not vary significantly among groups, a subgroup analysis in patients without esophageal varices showed that GR-MD-02 therapy reduced the hepatic venous pressure gradient and development of varices. Spasmodic cough was the only adverse event related to the study drug (NCT02462967) [224]. A phase III trial has been initiated to evaluate the safety and efficacy of GR-MD-02 in patients with NASH cirrhosis without esophageal varices (NCT04365868).

Promotion of a switch from a pathogenic to a restorative phenotype is an interesting strategy to accelerate fibrosis regression and promote liver regeneration [225]. This can be achieved by using pharmacological regulators that promote macrophage polarization. Steroids (e.g., dexamethasone), IL-4, IL-10, secretory leukocyte protease inhibitor (SLPI), prostaglandin E2 (PGE2) and colony-stimulating factor 1 receptor (CSF-1R) agonists have been explored for macrophage reprogramming in liver diseases [220]. Moreover, nanoparticles [226] represent a new approach that can selectively reprogram macrophages to a restorative phenotype [220].

Different types of nano systems have been developed for the recognition and targeting of macrophages, such as liposomes, solid-lipid, polymeric or metal nanoparticles. In order to design various nanoparticle systems, it is necessary to understand their mechanism of recognition by macrophages. Another controversial issue is the possible toxicity of non-degradable nanoparticles, which in many cases accumulate in the macrophages elimination organs such as the liver, spleen, and kidneys [226].

### 9.4. Exosome-Based Treatments

Exosomes are vesicles released by cells in both physiological and pathological conditions. They can contain distinct RNAs, proteins, lipids, and metabolites depending on the cell type of origin. Following their release into the intercellular milieu, exosomes bind to recipient cells and deliver their information which is then converted into epigenetic reprogramming driving phenotypic modifications [227]. Upon liver injury, exosomes released by epithelial cells deliver information able to activate fibroblasts, resulting in increased expression of α-SMA and type I collagen [228]. Endothelial cells release exosomes with LOXL2 located on the exterior side which increases collagen contraction [229]. Even aHSCs release exosomes containing CCN2, that contributes to the progression of fibrogenesis [230].

On the other hand, exosomes can be also involved in the regression of fibrosis [227]. Quiescent HSCs release exosomes which reduce HSC activation, while healthy hepatocytes secrete vesicles that can reduce the expression of profibrogenic genes. Alhomrani et al. identified human amnion epithelial cells secretoma as a negative modulator of liver fibrosis in CCl4 treated mice, acting on macrophage polarization, HSC activation and matrix deposition [231]. Mesenchymal stem cells (MSC)-derived exosomes are emerging as a potential tool to achieve liver fibrosis regression [232,233,234]. Exosomes released from human cord MSCs were found to ameliorate CCl_4_ -induced hepatic fibrosis in mice, while vesicles secreted by adipose tissue-derived MSCs were able to decrease the activation and proliferation of rat HSCs [233,234]. Along these lines, exosomes released in the serum could be employed as antifibrotic agents, as it was recently reported that circulating exosomes from healthy mice could reduce liver fibrosis in both CCl_4_- and TAA- mouse models [234]. In conclusion, “healthy exosomes”, containing “therapeutic factors”, emerge as a potential and powerful instrument for fibrotic liver treatment [234].

### 9.5. Mesenchymal Stem Cell Transplantation

Transplantation of stem cells, including MSCs, endothelial progenitors and haemopoietic stem cells, has proven to be effective in repairing fibrotic livers in experimental models, stimulating hepatocyte proliferation, inhibiting aHSCs, increasing MMP activity and inducing neovascularization [235]. MSCs have high proliferative capacity and multilineage potential and, when transplanted, migrate to fibrotic areas, and differentiate into hepatocyte-like cells or fuse with hepatocytes to restore liver function [235]. Human palatine tonsil-derived MSCs were shown to migrate to damaged livers but not to healthy livers. These cells, differentiating into hepatocyte-like cells, stimulated autophagy and decreased TGF-β signaling pathways, hampering liver fibrosis [236]. MSC transplantation was shown to improve hepatobiliary fibrosis, by inhibiting activation of HSCs, reducing collagen deposition, and increasing ECM degradation through an increase in MMP13 and a decrease of TIMP-1 [237,238].

Clinical trials have been conducted to test the efficacy of MSC transplantation, with controversial results. In cirrhotic patients MSCs were found to exert protective effects by increasing the amount of Treg cells and decreasing Th17 cells, leading to diminished serum levels of TGF-β, IL-17, TNF-α, and IL-6 [239]. In HBV-induced cirrhosis, MSC transfusion demonstrated to be clinically safe and to decrease ascites [240]. In other studies, MSC transplantation had no beneficial effects [241,242]. These discrepant results are probably due to the restricted number of patients enrolled and the short-term of follow up. Further investigation is warranted to elucidate the efficacy and the safety of this therapy for the treatment of fibrotic and cirrhotic patients. The main therapeutic approaches aimed to achieve the regression of fibrosis are summarized in Table 2.

### 9.6. CC Chemokine Receptor Antagonists

Chemokines coordinate inflammatory responses within different organs and induce the migration of fibrogenic cells to the sites of injury, thereby boosting fibrogenesis [110]. It has been observed that CCL5/RANTES, a ligand of chemokine receptor CCR5 induced by NFκB signaling, increased HSC migration and proliferation [243]. In experimental models of liver fibrosis (CCl4 and bile duct ligation), CCR1- and CCR5-knockout mice showed decreased hepatic fibrosis and macrophage infiltration [244]. An oral dual CCR2/CCR5 inhibitor, Cenicriviroc (CVC), showed anti-fibrotic effects in a thioacetamide-induced rodent model [245]. A phase II study using CVC, was conducted in NASH patients with liver fibrosis showing a beneficial effect of this agent even if not accompanied by anti-inflammatory action. Of note, treatment benefits were mostly shown in patients who showed higher fibrosis stage at baseline [246]. A phase III study (AURORA) in NASH patients with more advanced fibrosis was concluded early due to lack of efficacy of CVC based on the results of part I of the trial.

## 10. Conclusions

As well as fibrosis development, fibrosis regression is a complex and tightly regulated process that involves various cell types and several molecules, differently acting according to the changes in the ECM/inflammatory-driven microenvironment. Although reversal of fibrosis appears an encouraging approach to the treatment of chronic liver diseases, further studies are necessary to better understand the mechanisms underlying this process and to identify novel therapies for chronic liver disease. This could imply a decreased risk of developing hepatocellular carcinoma in patients affected by chronic liver disorders. In addition, while current therapies aimed to promote regression of fibrosis mainly focus on the removal of the noxious agents, there is need to deeper investigate on anti-fibrotic treatments able to positively modulate the mechanisms favoring fibrosis regression to confirm the long-term impact and strength of these findings. Indeed, even if some therapies or agents could represent promising tools to resolve hepatic fibrosis, none of the ones already used in human studies have been approved for clinical treatment.

## Figures and Tables

**Table 1:** Classification of human metalloproteinases (MMPs) and their function.

MMPs	GROUP	FUNCTION
MMP1, MMP8, MMP13	Collagenases	Cleavage of native fibrillar collagens to gelatin
MMP2, MMP9	Gelatinases	Degradation of a wide range of substrates, including gelatin, collagens and elastin
MMP12	Metalloelastases	Elastin degradation

**Table 2:** Major therapeutic approaches aimed to promote fibrosis regression.

THERAPY	TARGET(S)	MECHANISM(S) OF ACTION	PRE-CLINICAL OR CLINICAL STUDIES	STUDIES
LOXL2 inhibitors	Collagen and elastin cross-linking	Reduction of ECM stabilization and resistance to MMP degradation	Pre-clinical and clinical studies	[175,176,177]
Cilostazol	Phosphodiesterase III	Increase in intracellular cAMP with consequent inhibition of HSC and fibroblast activation	Pre-clinical studies	[189,190,191,192]
ET-1 receptor inhibitor	Endothelin-1 (ET-1)	Decrease in the contractile capacity of aHSCs mediated by interaction with LSEC and damaged hepatocytes	Pre-clinical and clinical studies	[196] (NCT03827200)
RAS inhibitor therapy (Candesartan)	TGFβ1	Reduction of liver fibrosis	Pre-clinical and clinical studies	[199] (NCT03770936)
Exosome-based treatments	Profibrogenic factors (α-SMA, TGFβ1)	Modulation of macrophage polarization, suppression of HSC activation and matrix deposition	Pre-clinical studies	[227,231,232,233,234]
Mesenchymal stem cell transplantation	Promote MSC migration into the fibrotic areas and their differentiation into hepatocyte-like cells to restore liver function	Stimulation of hepatocyte proliferation, reduction of HSC activation, increase in MMP activity and promotion of neovascularization	Pre-clinical and clinical studies	[232,235,236,237,238,239,240,241,242]

## Abbreviations

HSCs	hepatic stellate cells
ECM	extracellular matrix
NK	natural killer
CCl_4_	carbon tetrachloride
TAA	thioacetamide
BDL	bile duct ligation
EMT	epithelial-mesenchymal transition
MMPs	matrix metalloproteases
KCs	Kupffer cells
TIMPs	tissue inhibitors of metalloproteases
LSECs	liver sinusoidal endothelial cells
LOXL2	lysyl oxidase-like 2
RAS	renin-angiotensin system
MSCs	mesenchymal stem cells
cAMP	cyclic adenosine monophosphate
PKA	protein kinase A
CTGF	connective tissue growth factor
OCA	obeticholic acid
α-SMA	α-smooth muscle actin
SLPI	secretory leukocyte protease inhibitor
PGE2	prostaglandin E2
CSF-1R	colony-stimulating factor 1 receptor
TG	triacylglycerol
DAAs	direct-acting antivirals
Hh	Hedgehog
HIF	hypoxia-inducible factor
IL	interleukin
HCC	hepatocellular carcinoma
VEGF	vascular endothelial growth factor
